# The secure judgment of graphic similarity against malicious adversaries and its applications

**DOI:** 10.1038/s41598-023-30741-6

**Published:** 2023-03-21

**Authors:** Xin Liu, Yang Xu, Dan Luo, Gang Xu, Neal Xiong, Xiu-Bo Chen

**Affiliations:** 1 Computer Department, Tianjin Ren’ai College, Tianjin, 301636 China; 2 School of Information Engineering, Inner Mongolia university of science and technology, Baotou, 014010 China; 3 School of Information Science and Technology, North China University of Technology, Beijing, 100144 China; 4 Department of Computer Science and Mathematics, Sul Ross State University, Alpine, TX 79830 USA; 5 State Key Laboratory of Networking and Switching Technology, Beijing University of Posts and Telecommunications, Beijing, 100088 China

**Keywords:** Engineering, Mathematics and computing

## Abstract

With the advent of the era of big data, privacy computing analyzes and calculates data on the premise of protecting data privacy, to achieve data ‘available and invisible’. As an important branch of secure multi-party computation, the geometric problem can solve practical problems in the military, national defense, finance, life, and other fields, and has important research significance. In this paper, we study the similarity problem of geometric graphics. First, this paper proposes the adjacency matrix vector coding method of isomorphic graphics, and use the Paillier variant encryption cryptography to solve the problem of isomorphic graphics confidentiality under the semi-honest model. Using cryptography tools such as elliptic curve cryptosystem, zero-knowledge proof, and cut-choose method, this paper designs a graphic similarity security decision protocol that can resist malicious adversary attacks. The analysis shows that the protocol has high computational efficiency and has wide application value in terrain matching, mechanical parts, biomolecules, face recognition, and other fields.

## Introduction

At present, data have become a key strategic resource and are subverting the development model of the global society. However, data value and privacy protection are often in a state of opposition. Privacy computing technology has opened up a new model. On the premise of ensuring that the data provider does not disclose the original information, he uses and computes the data to realize data’s availability and invisibility. Privacy computing is widely used in blockchain^1–3^, sensor networks^4,5^, Identification of malicious and intrusion traffic in the Internet of Things^6,7^, and data collaborative computing^8,9^. Secure multi-party computation (MPC), one of three major technical tools of privacy computing, is a collaborative computing method used to compute various types of data. The research problems of MPC mainly include the following aspects: secure computational geometry^10–12^, secure data mining^13,14^, secure statistical analysis^15,16^, secure set relationship judgment^17,18^, etc.

As an important research branch of MPC, secure computing geometry can solve practical problems in the military^19^, national defense^20^, finance^21,22^, life^23^ and other fields, which has important research significance. Liu studied the secure computing distance between two points^11^. Zhang studied MPC of geometric area and volume^24^. Gong studied the MPC protocol of two private points composing a straight line^25^. In addition, secure computing geometry is widely used in the field of the positioning^26–28^. Among them, graphics retrieval technology is widely used in terrain matching^29^, mechanical parts retrieval^30^, biomolecular detection^23,31^, face recognition^32,33^, etc. So, it is particularly important to study the significance of secure judgment of graphic similarity.

In recent years, many cryptologists have made a research on graphic similarity. Reference^25^ proposed an MPC protocol of graphic similarity based on hash operations. Reference^34^ proposed an MPC protocol under the malicious model designed by ECC based on rational number equality. Reference^35^ solved the problem of determining the positional relationship between two rational intervals by using the inner product of an integer vector. But none of them can resist malicious behaviors.

The participants of MPC are divided into three categories: honest participants, semi-honest participants, and malicious participants. The malicious model is also designed based on the semi-honest model. Therefore, most of the studied MPC models in recent years are semi-honest models. Based on the study of the graphic isomorphic under the semi-honest model, a graphic similarity MPC protocol is proposed to resist the attacks of malicious enemies. The main contributions are as follows:

- Firstly, this paper analyzed the MPC judgment of graphic isomorphic under the semi-honest model based on the Paillier variant cryptosystem.
- This paper solves the potential malicious attacks in the protocol by using ECC, cut-choose method, and zero-knowledge proof, to design the graphic similarity MPC judgment protocol under the malicious model.
- Then, the correctness of the protocol is analyzed, and the computational performance is compared with existing protocols. The execution efficiency of the protocol is analyzed by experimental simulation. The security of the MPC protocol under the malicious model is proved by using the real/ideal paradigm method
- Finally, the practical value and research significance of the graphically similar confidentiality decision protocol are introduced. Protocol 2 proposed in this paper is widely used in terrain matching, mechanical parts, biomolecules, face recognition and other fields.

In this paper, section "Related knowledge" is the theoretical knowledge part, section "The MPC of graphic similarity under the semi-honest model" is the protocol design under the semi-honest model, section "The MPC of graphic similarity against malicious adversaries" is the protocol design under the malicious model, the correctness analysis of the protocol and the security proof. section "Efficiency analysis" is the experimental comparison and simulation of the protocol, and section "Applications" is the application of the protocol.

## Related knowledge

### Paillier variant cryptosystem

Paillier variant cryptosystem was proposed by Gong^25^, which can ensure the security of private data of participants without any key.

Key generation: two large prime $p$, $q$, compute $n = pq$, $\lambda = lcm(p - 1,q - 1)$. Public key is $n$, private key is $\lambda$.

Encryption: select random numbers $k \in Z_{n}$, $r_{1} \in Z_{n}$, $r_{2} \in Z_{n}$. Compute

1 $$g_{k} = (1 + n)^{k} \bmod n^{2} \,$$

2 $$c_{1} = g_{k}^{m} r_{1}^{n} \bmod n^{2}$$

3 $$c_{2} = g_{k}^{{}} r_{2}^{n} \bmod n^{2}$$

Decryption: $m = \frac{{L(c_{1}^{\lambda } {\text{mod}}n^{2} )}}{{L(c_{2}^{\lambda } {\text{mod}}n^{2} )}}\bmod n$ with the private key $\lambda$, where $L(u) = \frac{u - 1}{n}$.

Additive homomorphism:

4 $$E(x + y) = E(x) \cdot E(y)$$

5 $$E(x \cdot y) = E^{y} (x) = E^{x} (y)$$

Homomorphic operation:

6 $$\begin{gathered} c_{1}^{\omega } = (g_{k}^{m} r_{1}^{n} )^{\omega } \bmod n^{2} \\ = (1 + kn)^{\omega } r_{1}^{n \omega } \bmod n^{2} \\ = (1 + \omega kn)r_{1}^{n \omega } \bmod n^{2} \\ \end{gathered}$$

Securely compute the ratio of two numbers:

- (1) Alice randomly selects $k \in Z_{n}$, $r_{1} \in Z_{n}$, $r_{2} \in Z_{n}$, computes
  7 $$g_{k} = (1 + kn)\bmod n^{2}$$
  8 $$c_{1} = g_{k}^{y} r_{1}^{n} \bmod n^{2}$$
  9 $$c_{2} = g_{k}^{x} r_{2}^{n} \bmod n^{2}$$

for $x,y < n$, and sends the ciphertext $(c_{{1}} ,c_{{2}} )$ to Bob.

- (2) After Bob receives $(c_{{1}} ,c_{{2}} )$, he obtains $\frac{y}{x} = \frac{{L(c_{1}^{\lambda } \bmod n^{2} )}}{{L(c_{2}^{\lambda } \bmod n^{2} )}}$ through decryption.

### Elliptic curve cryptography

**Figure 1:**
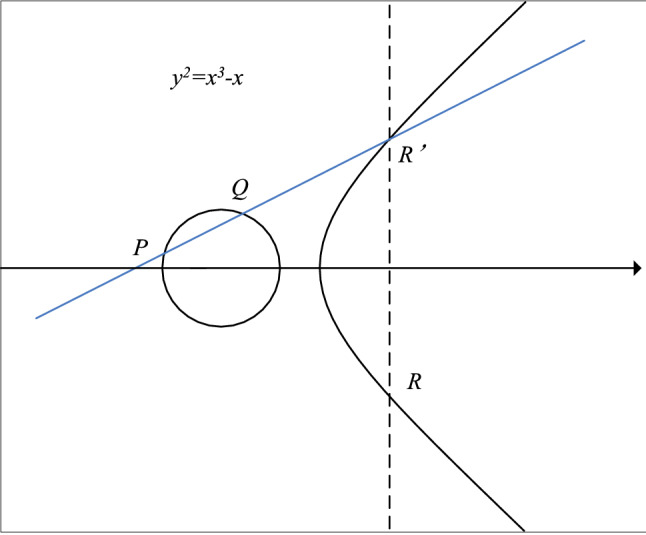
$$P + Q = R$$ in ECC.

Elliptic curve cryptography (ECC) is to determine an elliptic curve by both parties, pass through point $P$ and point $Q$ on $y^{2} = x^{3} - x$ to make a straight line intersecting any point $R^{\prime}$ on $y^{2} = x^{3} - x$, and then pass through point $R^{\prime}$ to make a straight line perpendicular to the $X$ axis and intersecting another point $R$ on $y^{2} = x^{3} - x$. we define its $P + Q = R$, as shown in Fig. 1 below:

When $P = Q$ in the above case, $R = 2P$. Then when $k$ identical $P$ are added, there is an operation: $k \cdot P$. ECC is to use the above operation to know that $k$ and $P$ can get $K = kP$; But on the contrary, knowing $P$ and $K$ cannot find $k$. That is, ECC uses $k$ as the private key and $K$ as the public key. The one-way solution ensures the safety of ECC.

Key generation: Select elliptic curve ${\rm E}_{p} (a,b)$, and determine a base point $G$, select private key $k$, then generate public key $K = kG$.

Encryption: Select a random number $r < n$ ($n$ is the order of point $G$), encode message $a$ to point $M$ on ${\rm E}_{p} (a,b)$, then calculate:

10 $$C_{1} = M + rK$$

11 $$C_{2} = rG$$

Decryption:

12 $$\begin{aligned} C_{1} - kC_{2} & = M + rK - k(rG) \\ & = M + r(K - kG) \\ & = M \\ \end{aligned}$$

Additive homomorphism:

Encryption: encode $m_{i}$ onto a point $M_{i}$ of ${\rm E}_{p} (a,b)$, use the private key $k$ to generate the public key $K = kG$, select a random number $r_{i}$, and compute $C_{1i} = M_{i} + rK$ and $C_{2i} = rG$.

Addition operation: $$\sum\limits_{i = 1}^{n} {C_{1i} } = C_{11} + C_{12} + ...C_{1n}$$, $$\sum\limits_{i = 1}^{n} {C_{2i} } = C_{21} + C_{22} + ...C_{2n}$$.

Decryption: compute:

13 $$\begin{gathered} \sum\limits_{i = 1}^{n} {C_{1i} - } k\sum\limits_{i = 1}^{n} {C_{2i} } = \sum\limits_{i = 1}^{n} {M_{i} } + K\sum\limits_{i = 1}^{n} {r_{i} } - kG\sum\limits_{i = 1}^{n} {r_{i} } \\ = \sum\limits_{i = 1}^{n} {M_{i} } \\ \end{gathered}$$

and finally decode $\sum\limits_{i = 1}^{n} {M_{i} }$ to obtain the plaintext $\sum\limits_{i = 1}^{n} {m_{i} }$.

### Security under the malicious model

To prove that the protocol is secure under the malicious model, it must be proved that it meets the security definition under the malicious model^36^. The security definition of the protocol under this malicious model was proposed by goldreich in 2009. If the actual protocol can achieve the same security as the ideal protocol, then this protocol is safe. In this article, In this article, Alice owns data x and Bob owns data y. They use the trusted third party (TTP) calculation function $f\left( {x,y} \right) = \left( {f_{1} \left( {x,y} \right),f_{2} \left( {x,y} \right)} \right)$ under the ideal model to compare the size. Through the trusted third party operation protocol, both parties finally get $f_{1} \left( {x,y} \right)$ and $f_{2} \left( {x,y} \right)$ without leaking $x$ and $y$. This proves that this protocol is safe under the malicious model. The specific analysis is as follows:

Ideas: Alice and Bob have data $x$ and $y$ respectively. They compute

14 $$f\left( {x,y} \right) = \left( {f_{1} \left( {x,y} \right),f_{2} \left( {x,y} \right)} \right)$$

with the help of trusted third party (TTP). After the execution of the protocol, both parties get $f_{1} \left( {x,y} \right)$ and $f_{2} \left( {x,y} \right)$ respectively, but do not disclose $x$ and $y$. The steps are as follows:

- (1) Alice’s and Bob’s inputs $x$ and $y$ respectively.
- (2) The honest party will always provide the correct $x$, $y$ to TTP. Malicious participants may decide not to execute the protocol, or provide wrong $x^{\prime}$ or $y^{\prime}$ when executing the protocol.
- (3) After the TTP obtains the inputs $\left( {x,y} \right)$, he computes $f\left( {x,y} \right)$ and sends $f_{1} \left( {x,y} \right)$ to Alice, otherwise he sends a special symbol $\bot$ to Alice.
- (4) If Alice is a malicious participant, it may ignore the TTP after receiving $f_{1} \left( {x,y} \right)$. At this time, TTP sends $\bot$ to Bob; otherwise, TTP will send $f_{2} \left( {x,y} \right)$ to Bob.

Because both participants cannot get other information except their own $f_{i} \left( {x,y} \right)$ from TTP, the ideal model protocol is secure. If the real model protocol can achieve the same security, then the real model protocol is secure.

In the ideal model protocol, participants have auxiliary information $z$ and take the process of jointly calculating $F(x,y)$ by strategy $\overline{B}$ as $IDEAL_{{F,\overline{B} (z)}} (x,y)$, which is defined as that the enemy evenly selects a random number $r$ to make $IDEAL_{{F,\overline{B} (z)}} (x,y) = \gamma (x,y,z,r)$, where $\gamma (x,y,z,r)$ is defined as follows (note: if both parties are malicious, it is impossible to design a secure protocol, which will not be considered. Protocol 2 under the malicious model is a protocol that can resist malicious attacks by default):

- If Alice is honest, then
  15 $$\gamma (x,y,z,r) = (f_{1} (x,y^{^{\prime}} ),B_{2} (y,z,r,f_{2} (x,y^{^{\prime}} )))$$

where $$y^{^{\prime}} = B_{2} (y,z,r)$$.

- If Bob is honest,
  16 $$\gamma (x,y,z,r) = \left\{ {\begin{array}{*{20}c} {(B_{1} (x,z,r,f_{1} (x^{{\prime}} ,y), \bot ), \bot ),} \\ {if \quad B_{1} (x,z,r,f_{1} (x^{{\prime}} ,y)) = \bot ;} \\ {(B_{1} (x,z,r,f_{1} (x^{{\prime}} ,y)),f_{2} (x^{{\prime}} ,y)),} \\ {otherwise.} \\ \end{array} } \right.$$

In both cases, $x^{{\prime}} = B_{1} (x,z,r)$.

#### Definition 1

Security of the malicious model protocol.

If an acceptable policy pair $\overline{A} = (A_{1} ,A_{2} )$ can be found in the real model protocol $\Pi$, there is an acceptable policy pair $\overline{B} = (B_{1} ,B_{2} )$ in the ideal model protocol, making the

17 $$\{ IDEAL_{{F,\overline{B} (z)}} (x,y)\}_{x,y,z} \mathop \equiv \limits^{c} \{ REAL_{{\Pi ,\overline{A} (z)}} (x,y)\}_{x,y,z}$$

That is to say the protocol $\Pi$ securely computes $F$.

## The MPC of graphic similarity under the semi-honest model

### Solutions

Problem description: Alice has a graphic $O$ and Bob has a graphic $P$, both parties want to determine whether the graphics $O$ and $P$ are isomorphic without disclosing any information.

Ideas: The Protocol 1 uses the Paillier variant cryptosystem proposed in Reference^25^. First, the criterion for judging the isomorphism of two graphics is whether the adjacency matrices of two graphics are equal. Alice encodes her graphic, marks the starting point of the longest edge as 1, numbers the vertices in turn in a counterclockwise direction, and computes her adjacency matrix $M_{1}$ (that is, there are edges between the vertices, the elements in the matrix are marked as 1, the non existence is marked as 0, and the self vertex is marked as 0). At the same time, Bob encodes his graphics counterclockwise and clockwise respectively (still taking the longest edge as the starting point), and computes the adjacency matrices $M_{2}$ and $M^{\prime}_{2}$ twice. If two graphics are isomorphic, then $M_{1} { = }M_{2}$ or $M_{1} { = {{\rm M}^{\prime}}}_{{2}}$.

**Figure 2:**
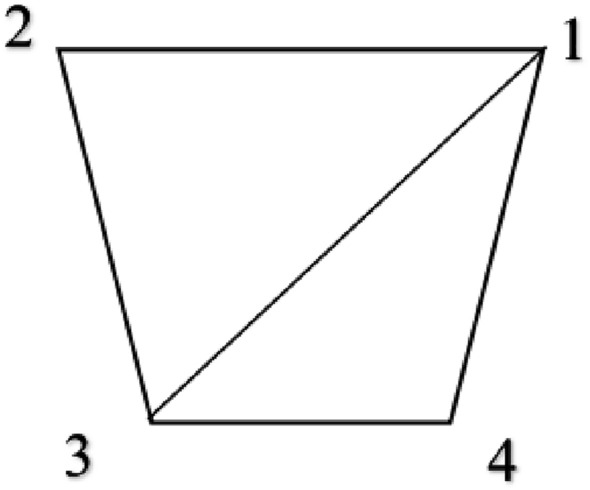
Matrix example.

**Figure 3:**
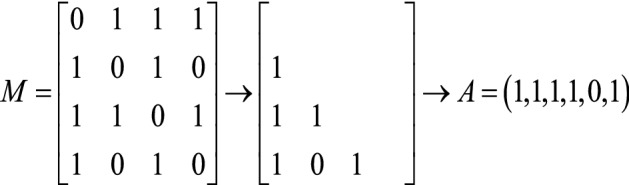
Vector of adjacency matrix.

Because the adjacency matrices are composed of 0 and 1, and the adjacency matrices are undirected graphics with diagonal elements of 0, we can select the data of the lower triangle of the matrix for vector arrangement to obtain the vector $A$ (as shown in Figs. 2 and 3).

**Figure 4:**
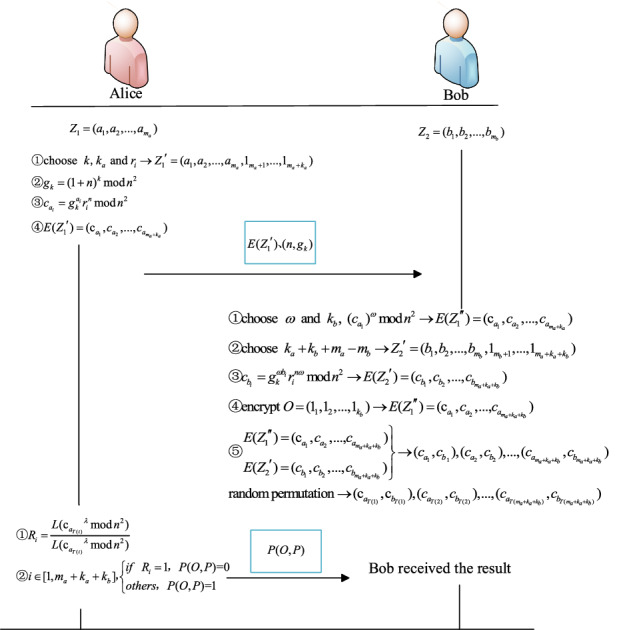
Protocol flow chart under the semi-honest model.

The flow chart of the MPC protocol for isomorphic graphics under the semi-honest model is shown in Fig. 4. Alice encrypts the vector and sends it to Bob. Bob performs ciphertext operation between his encrypted vector and the received ciphertext, returns the result of the operation to Alice, and then decrypts it to get the result. See Protocol 1 for the specific protocol.

### Specific protocol

Protocol 1 uses the Paillier variant cryptosystem to securely compute the ratio of two numbers, and then securely judge the isomorphism of two graphics.

**Table:** 

Protocol 1: The MPC of graphic similarity under the semi-honest model	
**Input:** Alice owns a graphic $O$ and Bob owns a graphic $P$ **Output:** $$P(O,P) = \left\{ {\begin{array}{*{20}l} 0, & {O\;and\;P\;are\;\, isomorphism}\\ 1, & {O\;and\;P\;are\;not\;isomorphism}\end{array} } \right.$$ **Preparation:** Alice generates public key $n$ and private key $\lambda$. Alice constructs a vector according to the above method to obtain $Z_{1} = {(}a_{1} ,a_{2} ,...,a_{{m_{a} }} {)}$. At the same time, Bob constructs vectors to obtain $Z_{2} = (b_{1} ,b_{2} ,...,b_{{m_{b} }} )$ and $Z^{\prime}_{2} = (b^{\prime}_{1} ,b^{\prime}_{2} ,...,b^{\prime}_{{m_{b} }} )$ **Start:** • (1) Alice selects the random numbers $k$, $k_{a}$ and $r_{i}$, and adds $k_{a}$ 1s to the last of $Z_{1} = {(}a_{1} ,a_{2} ,...,a_{{m_{a} }} {)}$ to get $Z_{1}^{\prime } = {(}a_{1} ,a_{2} ,...,a_{{m_{a} }} ,1_{{m_{a} + 1}} ,...,1_{{m_{a} + k_{a} }} )$. She computes $g_{k} = (1 + n)^{k} \bmod n^{2}$, encrypts $Z_{1}^{\prime }$ with the public key $n$, that is, computes $c_{{a_{i} }} = g_{k}^{{a_{i} }} r_{i}^{n} \bmod n^{2}$ to obtain $E(Z_{1}^{\prime } ) = {\text{(c}}_{{a_{1} }} ,c_{{a_{2} }} ,...,c_{{a_{{m_{a} + k_{a} }} }} {)}$. Send $E(Z_{1}^{\prime } )$ and $(n,g_{k} )$ to Bob • (2) Bob selects the random numbers $\omega$ and $k_{b}$ and performs homomorphic operation on the elements of $E(Z_{1}^{\prime } ) = {\text{(c}}_{{a_{1} }} ,c_{{a_{2} }} ,...,c_{{a_{{m_{a} + k_{a} }} }} {)}$, and $E(Z_{1}^{\prime \prime } ) = {{({\rm c}^{\prime}}}_{{a_{1} }} ,c^{\prime}_{{a_{2} }} ,...,c^{\prime}_{{a_{{m_{a} + k_{a} }} }} {)}$, in which ${{{\rm c}^{\prime}}}_{{a_{i} }} = (c_{{a_{i} }} )^{\omega } \bmod n^{2} = (g_{k}^{{a_{i} }} r_{i}^{n} )^{\omega } \bmod n^{2} = (1 + n)^{{\omega }{a_{i} }} r_{i}^{n \omega } \bmod n^{2} = (1 + \omega n)^{{a_{i} }} r_{i}^{n \omega } \bmod n^{2}$.Select the random number $k_{a} + k_{b} + m_{a} - m_{b}$ and add $k_{a} + k_{b} + m_{a} - m_{b}$ 1s to the last of $Z_{2} = (b_{1} ,b_{2} ,...,b_{{m_{b} }} )$ to get $Z_{2}^{\prime } = {(}b_{1} ,b_{2} ,...,b_{{m_{b} }} ,1_{{m_{b} + 1}} ,...,1_{{m_{a} + k_{a} + k_{b} }} )$. Use a random number $\omega$ and public key $n$ to encrypt vector $Z_{2}^{\prime } = {(}b_{1} ,b_{2} ,...,b_{{m_{b} }} ,1_{{m_{b} + 1}} ,...,1_{{m_{a} + k_{a} + k_{b} }} )$, obtains $E(Z_{2}^{\prime } ) = (c_{{b_{1} }} ,c_{{b_{2} }} ,...,c_{{b_{{m_{a} + k_{a} + k_{b} }} }} )$, in which $c_{{b_{i} }} = g_{k}^{{\omega b_{i} }} r_{i}^{n\omega } \bmod n^{2} = (1 + kn)^{{\omega }{b_{i} }} r_{i}^{n\omega } \bmod n^{2} = (1 + \omega kn)^{{b_{i} }} r_{i}^{n \omega } \bmod n^{2}$. Arrange $k_{b}$ 1s into the vector $O = (1_{1} ,1_{2} ,...,1_{{k_{b} }} )$ and encrypt vector $O = (1_{1} ,1_{2} ,...,1_{{k_{b} }} )$ to obtain: $E(O) = (c_{{1_{1} }} ,c_{{1_{2} }} ,...,c_{{1_{{k_{b} }} }} )$. Add $E(O)$ to the last of $E(Z_{1}^{\prime \prime } ) = {{({\rm c}^{\prime}}}_{{a_{1} }} ,c^{\prime}_{{a_{2} }} ,...,c^{\prime}_{{a_{{m_{a} + k_{a} }} }} {)}$ to get a $E(Z_{1} ^{\prime\prime\prime}) = {\text{(c}}_{{a_{1} }} ,c_{{a_{2} }} ,...{,}c_{{a_{{m_{a} + k_{a} + k_{b} }} }} {)}$. Combine $E(Z_{1} ^{\prime\prime \prime}) = {\text{(c}}_{{a_{1} }} ,c_{{a_{2} }} ,...{,}c_{{a_{{m_{a} + k_{a} + k_{b} }} }} {)}$ and $E(Z_{2}^{\prime } ) = (c_{{b_{1} }} ,c_{{b_{2} }} ,...,c_{{b_{{m_{a} + k_{a} + k_{b} }} }} )$ into the vector group $(c_{{a_{1} }} ,c_{{b{}_{1}}} ),(c_{{a_{2} }} ,c_{{b_{2} }} ),...,(c_{{a_{{m_{a} + k_{a} + k_{b} }} }} ,c_{{b_{{m_{a} + k_{a} + k_{b} }} }} )$ in turn, randomly shuffles their order to obtain ${\text{(c}}_{{a_{T(1)} }} ,{\text{c}}_{{b_{T(1)} }} ),(c_{{a_{T(2)} }} ,c_{{b_{T(2)} }} ),...,(c_{{a_{{T(m_{a} + k_{a} + k_{b} )}} }} ,c_{{b_{{T(m_{a} + k_{a} + k_{b} )}} }} {)}$, and send them to Alice • (3) Alice uses the private key $\lambda$ to decrypt each group of ciphertext in ${\text{(c}}_{{a_{T(1)} }} ,{\text{c}}_{{b_{T(1)} }} ),(c_{{a_{T(2)} }} ,c_{{b_{T(2)} }} ),...,(c_{{a_{{T(m_{a} + k_{a} + k_{b} )}} }} ,c_{{b_{{T(m_{a} + k_{a} + k_{b} )}} }} {)}$: $R_{i} = \frac{{L({\text{c}}_{{a_{T(i)} }}^{\lambda } \bmod n^{2} )}}{{L({\text{c}}_{{b_{T(i)} }}^{\lambda } \bmod n^{2} )}}$. For any $i \in [1,m_{a} + k_{a} + k_{b} ]$, if $R_{i} = 1$, $P(O,P){ = }0$; otherwise, $P(O,P){ = }1$. Similarly, it can be judged whether the other vector $Z^{\prime}_{2} = (b^{\prime}_{1} ,b^{\prime}_{2} ,...,b^{\prime}_{{m_{b} }} )$ of Bob and the vector $Z_{1} = {(}a_{1} ,a_{2} ,...,a_{{m_{a} }} {)}$ of Alice are isomorphic matrices **The protocol ends**	

In the above semi-honest model protocol, the participants may carry out some malicious attacks. Therefore, a judgment protocol of graphic similarity against malicious adversaries is proposed to improve the security in the following section.

## The MPC of graphic similarity against malicious adversaries

### Solutions

Protocol 1 only solves the problem of graphic isomorphism, and cannot resist the attacks of malicious enemies. Based on the MPC of graphic isomorphism, using the cryptography tools to solve the problem of graphic similarity, a MPC protocol of graphic similarity is proposed to resist or prevent the possible malicious attacks.

Rules for judging the similarity of two graphics: If two graphics are isomorphic and the corresponding angles are equal, the two graphics are similar. Then, based on the MPC of the graphic isomorphism in Protocol 1, the judgment of corresponding angles is added to judge whether the two graphics are similar.

We first analyze Protocol 1 under the semi-honest model, and use our cryptography tools to solve the possible malicious behavior in this protocol. Ideally, malicious acts that cannot be prevented in any way are: both parties refuse to participate in the agreement, one party of both parties inputs false information (This situation includes that one of the parties does not use the same rules to construct the adjacency matrix), and one party of the agreement will not participate in the next agreement after receiving the information. In addition, the potential malicious behaviors of Protocol 1 include:

- Alice has both a public key and a private key, but Bob has only a public key, and the output of the protocol can only be decrypted by Alice, which is very unfair to Bob.
- Alice told Bob the wrong protocol output.
- During the process of the protocol, Alice transmits the wrong ciphertext to Bob, resulting in the wrong result for both parties at the end of the protocol.

We must solve the above problems and design protocols that can resist malicious attacks.

(1) Alice constructs the isomorphic matrix vector $Z_{1} = {(}a_{1} ,a_{2} ,...,a_{m} {)}$ according to the method in Fig. 2, and sorts it diagonally counterclockwise to obtain angular vector $D_{1} = (c_{1} ,c_{2} ,...,c_{n} )$ ($c_{i}$ retains two decimal places and expands it by 100 times).

(2) Bob makes the above coding for his graphic counterclockwise and clockwise, and obtains two groups of isomorphic matrix vectors $Z_{2} = (b_{1} ,b_{2} ,...,b_{m} )$ and $Z^{\prime}_{2} = (b^{\prime}_{1} ,b^{\prime}_{2} ,...,b^{\prime}_{m} )$, angular vectors $D_{2} = (d_{1} ,d_{2} ,...,d_{n} )$ and $D^{\prime}_{2} = (d^{\prime}_{1} ,d^{\prime}_{2} ,...,d^{\prime}_{n} )$ respectively. If $((Z_{1} = Z_{2} ) \vee (Z_{1} = Z^{\prime}_{2} )) \wedge ((D_{1} = D_{2} ) \vee (D_{1} = D^{\prime}_{2} ))$, the two graphics are similar.

**Figure 5:**
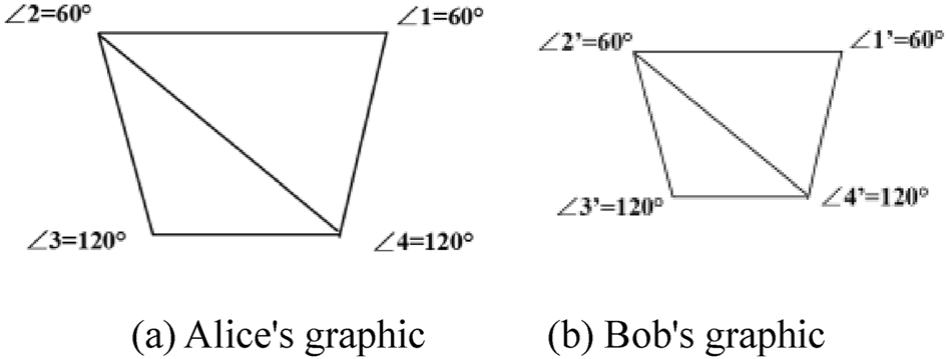
Example of similar graphics.

Alice connects the matrix vector $Z_{1} = {(}a_{1} ,a_{2} ,...,a_{m} {)}$ with the angle vector $D_{1} = (c_{1} ,c_{2} ,...,c_{n} )$ to obtain the vector $A = {(}a_{1} ,a_{2} ,...,a_{m} {,}c_{1} ,c_{2} ,...,c_{n} {)}$. Similarly, Bob gets two vectors $B = {(}b_{1} ,b_{2} ,...,b_{m} {,}d_{1} ,d_{2} ,...,d_{n} {)}$ and $B^{\prime} = {(}b^{\prime}_{1} ,b^{\prime}_{2} ,...,b^{\prime}_{m} {,}d^{\prime}_{1} ,d^{\prime}_{2} ,...,d^{\prime}_{n} {)}$. As shown in Fig. 5, Alice and Bob each have a quadrilateral graphic (a) and graphic (b). In (a), Alice's matrix vector is $Z_{1} = \left( {1,1,1,1,0,1} \right)$ and the angle vector $D_{1} = (60,60,120,120)$ to obtain vector $A = {(}1,1,1,1,0,1{,}60,60,120,120{)}$. Bob gets vector $B = {(}1,1,1,1,0,1{,}60,60,120,120{)}$ in the same way. If the two vectors are equal, the two graphics are similar. Similarly, the hash function can be used to directly judge whether matrices *A* and *B* are equal. However, if the result is unequal, there is no way to distinguish whether it is unequal or one of the participants is a malicious participant. Therefore, the method of using hash function is not feasible.

**Figure 6:**
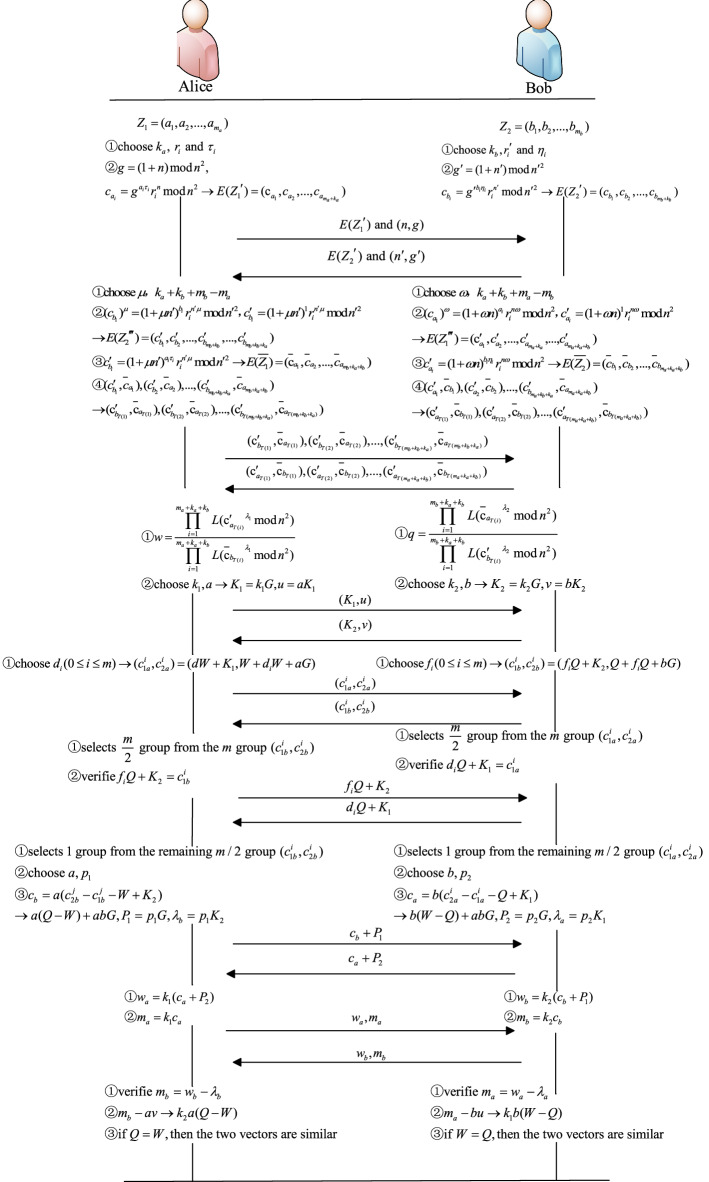
Protocol flow chart under the malicious model.

The flow chart of the graphically similar confidentiality decision protocol under the malicious model is shown in Fig. 6 both parties perform each step of the protocol fairly and fairly. With the help of cryptography tools such as ECC, zero-knowledge proof and split-selection method, the possible malicious behaviors in Protocol 1 are prevented, and the possible malicious behaviors in Protocol 1 are solved one by one. Finally, the malicious behaviors can not be implemented or are found when the malicious behaviors are implemented, and the final results are calculated by both parties at the same time.

### Specific protocol

Using the above vector coding rules, with the help of ECC, zero-knowledge proof and cut-choose method, a MPC protocol of graphic similarity against malicious attacks is proposed.

**Table:** 

Protocol 2: The MPC protocol of graphic similarity against malicious attacks	
**Input: Alice owns a graphic **$O$** and Bob owns a graphic **$P$ **Output: **$$P(O,P) = \left\{ {\begin{array}{*{20}l} 0, & {O\;and\;P\;are\;\, isomorphism}\\ 1, & {O\;and\;P\;are\;not\;isomorphism}\end{array} } \right.$$ **Preparation:** Alice generates public key $n$ and private key $\lambda$. Alice constructs a vector according to the above method to obtain $Z_{1} = {(}a_{1} ,a_{2} ,...,a_{{m_{a} }} {)}$. At the same time, Bob constructs vectors to obtain $Z_{2} = (b_{1} ,b_{2} ,...,b_{{m_{b} }} )$ and $Z^{\prime}_{2} = (b^{\prime}_{1} ,b^{\prime}_{2} ,...,b^{\prime}_{{m_{b} }} )$ **Start:** • (1) Alice selects random prime numbers $k_{a}$, $r_{i}$ and $\tau_{i} (1 \le i \le m_{a} )$, computes $g = (1 + n)\bmod n^{2}$, encrypts vector $Z_{1} = {(}a_{1} ,a_{2} ,...,a_{{m_{a} }} {)}$, that is, obtains $E(Z_{1} ) = {\text{(c}}_{{a_{1} }} ,c_{{a_{2} }} ,...,c_{{a_{{m_{a} }} }} {)}$, in which $c_{{a_{i} }} = g_{{}}^{{a_{i} \tau_{i} }} r_{i}^{n} \bmod n^{2}$. At the same time, the public key $n$ is used to encrypt $k_{a}$ 1s, that is, to compute $c_{{a_{i} }} = g_{{}}^{{a_{i} }} r_{i}^{n} \bmod n^{2}$, and the computed vector is placed behind $E(Z_{1} ) = {\text{(c}}_{{a_{1} }} ,c_{{a_{2} }} ,...,c_{{a_{{m_{a} }} }} {)}$ to obtain a new vector $E(Z_{1}^{\prime } ) = {\text{(c}}_{{a_{1} }} ,c_{{a_{2} }} ,...,c_{{a_{{m_{a} + k_{a} }} }} {)}$. Send $E(Z_{1}^{\prime } )$ and $(n,g)$ to Bob • (2) Bob selects random prime numbers $k_{b}$, $r_{i}^{\prime }$ and $\eta_{i} (1 \le i \le m_{b} + k_{b} )$, computes $g^{\prime} = (1 + n^{\prime})\bmod n^{{\prime}{2}}$, encrypts vector $Z_{2} = (b_{1} ,b_{2} ,...,b_{{m_{b} }} )$, that is, obtains $E(Z_{2} ) = (c_{{b_{1} }} ,c_{{b_{2} }} ,...,c_{{b_{{m_{b} }} }} )$, in which $c_{{b_{i} }} = g^{\prime b_{i} \eta_{i} } r_{i}^{\prime n \prime} \bmod n^{{\prime}{2}}$. At the same time, $k_{b}$ 1s are encrypted by $g^{\prime}$ and public key $n^{\prime}$, that is, $c_{{b_{i} }} = g^{\prime b_{i} } r^{\prime n \prime}_{i} \bmod n^{{\prime}{2}}$ is computed, and the computed vector is placed behind $E(Z_{2} ) = (c_{{b_{1} }} ,c_{{b_{2} }} ,...,c_{{b_{{m_{b} }} }} )$ to obtain a new vector $E(Z_{2}^{\prime } ) = (c_{{b_{1} }} ,c_{{b_{2} }} ,...,c_{{b_{{m_{b} + k_{b} }} }} )$. Send $E(Z_{2}^{\prime } )$ and $(n^{\prime},g^{\prime})$ to Alice • (3) Alice selects the random number $\mu$ and performs homomorphic operation on the elements of $E(Z_{2}^{\prime } ) = (c_{{b_{1} }} ,c_{{b_{2} }} ,...,c_{{b_{{m_{b} + k_{b} }} }} )$ to obtain $E(Z_{2}^{\prime \prime } ) = (c^{\prime}_{{b_{1} }} ,c^{\prime}_{{b_{2} }} ,...,c^{\prime}_{{b_{{m_{b} + k_{b} }} }} )$, where $(c_{{b_{i} }} )^{\mu } = (1 + \mu n^{\prime})^{{b_{i} }} r_{i}^{\prime n \prime \mu } \bmod n^{{\prime}{2}}$. Then, the random number $\mu$ and public key $n^{\prime}$ are used to encrypt $k_{a}$ 1s, that is, $c^{\prime}_{{b_{i} }} = (1 + \mu n^{\prime})^{1} r_{i}^{\prime n \prime \mu } \bmod n^{{\prime}{2}}$, and the computed vector is placed behind $E(Z_{2}^{\prime \prime } ) = (c^{\prime}_{{b_{1} }} ,c^{\prime}_{{b_{2} }} ,...,c^{\prime}_{{b_{{m_{b} + k_{b} }} }} )$ to obtain a new vector $E(Z_{2}^{\prime \prime \prime } ) = (c^{\prime}_{{b_{1} }} ,c^{\prime}_{{b_{2} }} ,...,c^{\prime}_{{b_{{m_{b} + k_{b} }} }} ,...,c^{\prime}_{{b_{{m_{b} + k_{b} + k_{a} }} }} )$. Select the random number $k_{a} + k_{b} + m_{b} - m_{a}$, add $k_{a} + k_{b} + m_{b} - m_{a}$ 1s to the last of $Z_{1} = {(}a_{1} ,a_{2} ,...,a_{{m_{a} }} {)}$, and encrypt the $m_{b} + k_{a} + k_{b}$ vectors by using the random number $\mu$, $\tau_{i} (1 \le i \le m_{a} )$ and public key $n^{\prime}$, that is, compute $\overline{c}_{{b_{i} }} = (1 + \mu n^{\prime})^{{a_{i} \tau_{i} }} r_{i}^{{n{\prime \mu }}} \bmod n^{{\prime}{2}}$ to obtain $E(\overline{{Z_{1} }} ) = {(}\overline{{\text{c}}}_{{a_{1} }} ,\overline{c}_{{a_{2} }} ,...,\overline{c}_{{a_{{m_{b} + k_{a} + k_{b} }} }} {)}$. Construct a one-to-one array of $E(Z_{2}^{\prime \prime \prime } ) = (c^{\prime}_{{b_{1} }} ,c^{\prime}_{{b_{2} }} ,...,c^{\prime}_{{b_{{m_{b} + k_{b} }} }} ,...,c^{\prime}_{{b_{{m_{b} + k_{b} + k_{a} }} }} )$ and $E(\overline{{Z_{1} }} ) = {(}\overline{{\text{c}}}_{{a_{1} }} ,\overline{c}_{{a_{2} }} ,...,\overline{c}_{{a_{{m_{b} + k_{a} + k_{b} }} }} {)}$ in order: $(c^{\prime}_{{b_{1} }} ,\overline{c}_{{a_{1} }} ),(c^{\prime}_{{b_{2} }} ,\overline{c}_{{a_{2} }} ),...,(c^{\prime}_{{b_{{m_{b} + k_{b} + k_{a} }} }} ,\overline{c}_{{a_{{m_{b} + k_{a} + k_{b} }} }} )$. Alice disrupts the order to get ${{({\rm c}^{\prime}}}_{{b_{T(1)} }} ,\overline{{\text{c}}}_{{a_{T(1)} }} ),({{{\rm c}^{\prime}}}_{{b_{T(2)} }} ,\overline{{\text{c}}}_{{a_{T(2)} }} ),...,({{{\rm c}^{\prime}}}_{{b_{{T(m_{b} + k_{b} + k_{a} )}} }} ,\overline{{\text{c}}}_{{a_{{T(m_{b} + k_{b} + k_{a} )}} }} {)}$, and sends it to Bob • (4) Bob selects the random number $\omega$ and performs homomorphic operation on the elements of $E(Z_{1}^{\prime } ) = {\text{(c}}_{{a_{1} }} ,c_{{a_{2} }} ,...,c_{{a_{{m_{a} + k_{a} }} }} {)}$ to obtain $E(Z_{1}^{\prime \prime } ) = {{({\rm c}^{\prime}}}_{{a_{1} }} ,c^{\prime}_{{a_{2} }} ,...,c^{\prime}_{{a_{{m_{a} + k_{a} }} }} {)}$, in which $(c_{{a_{1} }} )^{\omega } = (1 + \omega n)^{{a_{i} }} r_{i}^{n \omega } \bmod n^{2}$. Then, $k_{b}$ 1s are encrypted by random number $\omega$ and public key $n$, that is, $c^{\prime}_{{a_{i} }} = (1 + \omega n)^{1} r_{i}^{n\omega } \bmod n^{2}$. The computed vector is placed after $E(Z_{1}^{\prime \prime } ) = {{({\rm c}^{\prime}}}_{{a_{1} }} ,c^{\prime}_{{a_{2} }} ,...,c^{\prime}_{{a_{{m_{a} + k_{a} }} }} {)}$ to obtain a new vector $E(Z_{1}^{\prime \prime \prime } ) = {{({\rm c}^{\prime}}}_{{a_{1} }} ,c^{\prime}_{{a_{2} }} ,...,c^{\prime}_{{a_{{m_{a} + k_{a} }} }} {,}...{,}c^{\prime}_{{a_{{m_{a} + k_{a} + k_{b} }} }} {)}$. Select the random number $k_{a} + k_{b} + m_{a} - m_{b}$ and add the $k_{a} + k_{b} + m_{a} - m_{b}$ 1s to the last of $Z_{2} = (b_{1} ,b_{2} ,...,b_{{m_{b} }} )$ to get $\overline{{Z_{2} }} = {(}b_{1} ,b_{2} ,...,b_{{m_{b} }} ,1_{{m_{b} + 1}} ,...,1_{{m_{a} + k_{a} + k_{b} }} )$. The random numbers $\omega$, $\eta_{i} (1 \le i \le m_{b} + k_{b} )$ and public key $n$ are used to encrypt the $m_{a} + k_{a} + k_{b}$ vectors, that is, $\overline{c}_{{a_{i} }} = (1 + \omega n)^{{b_{i} \eta_{i} }} r^{\prime n\omega }_{i} \bmod n^{2}$ is computed to obtain $E(\overline{{Z_{2} }} ) = (\overline{c}_{{b_{1} }} ,\overline{c}_{{b_{2} }} ,...,\overline{c}_{{b_{{m_{a} + k_{a} + k_{b} }} }} )$. Construct a one-to-one array of $E(Z_{1}^{\prime \prime \prime } ) = {{({\rm c}^{\prime}}}_{{a_{1} }} ,c^{\prime}_{{a_{2} }} ,...,c^{\prime}_{{a_{{m_{a} + k_{a} }} }} {,}...{,}c^{\prime}_{{a_{{m_{a} + k_{a} + k_{b} }} }} {)}$ and $E(\overline{{Z_{2} }} ) = (\overline{c}_{{b_{1} }} ,\overline{c}_{{b_{2} }} ,...,\overline{c}_{{b_{{m_{a} + k_{a} + k_{b} }} }} )$ in order: $(c^{\prime}_{{a_{1} }} ,\overline{c}_{{b_{1} }} ),(c^{\prime}_{{a_{2} }} ,\overline{c}_{{b_{2} }} ),...,(c^{\prime}_{{b_{{m_{a} + k_{b} + k_{a} }} }} ,\overline{c}_{{a_{{m_{a} + k_{a} + k_{b} }} }} )$. And Bob disrupts the order ${{({\rm c}^{\prime}}}_{{a_{T(1)} }} ,\overline{{\text{c}}}_{{b_{T(1)} }} ),({{{\rm c}^{\prime}}}_{{a_{T(2)} }} ,\overline{{\text{c}}}_{{b_{T(2)} }} ),...,({{{\rm c}^{\prime}}}_{{a_{{T(m_{a} + k_{a} + k_{b} )}} }} ,\overline{{\text{c}}}_{{b_{{T(m_{a} + k_{a} + k_{b} )}} }} {)}$ and sends it to Alice • (5) Alice uses the private key $\lambda_{1}$ to decrypt each element in ${{({\rm c}^{\prime}}}_{{a_{T(1)} }} ,\overline{{\text{c}}}_{{b_{T(1)} }} ),({{{\rm c}^{\prime}}}_{{a_{T(2)} }} ,\overline{{\text{c}}}_{{b_{T(2)} }} ),...,({{{\rm c}^{\prime}}}_{{a_{{T(m_{a} + k_{a} + k_{b} )}} }} ,\overline{{\text{c}}}_{{b_{{T(m_{a} + k_{a} + k_{b} )}} }} {)}$, that is $w = \frac{{\prod\limits_{i = 1}^{{m_{a} + k_{a} + k_{b} }} {L({{{\rm c}^{\prime}}}_{{a_{T(i)} }}^{{\lambda_{1} }} \bmod n^{2} )} }}{{\prod\limits_{i = 1}^{{m_{a} + k_{a} + k_{b} }} {L(\overline{{\text{c}}}_{{b_{T(i)} }}^{{\lambda_{1} }} \bmod n^{2} )} }}$ • (6) Bob uses the private key $\lambda_{2}$ to decrypt each element in ${{({\rm c}^{\prime}}}_{{b_{T(1)} }} ,\overline{{\text{c}}}_{{a_{T(1)} }} ),({{{\rm c}^{\prime}}}_{{b_{T(2)} }} ,\overline{{\text{c}}}_{{a_{T(2)} }} ),...,({{{\rm c}^{\prime}}}_{{b_{{T(m_{b} + k_{b} + k_{a} )}} }} ,\overline{{\text{c}}}_{{a_{{T(m_{b} + k_{b} + k_{a} )}} }} {)}$, that is $q = \frac{{\prod\limits_{i = 1}^{{m_{b} + k_{a} + k_{b} }} {L(\overline{{\text{c}}}_{{a_{T(i)} }}^{{\lambda_{2} }} \bmod n^{2} )} }}{{\prod\limits_{i = 1}^{{m_{b} + k_{a} + k_{b} }} {L({{{\rm c}^{\prime}}}_{{b_{T(i)} }}^{{\lambda_{2} }} \bmod n^{2} )} }}$ • (7) Both parties select an elliptic curve $w$ and a base point $w$ at the same time; Then select their own private key $k_{1}$, $k_{2}$, generate their own public key $w$ and $w$ according to the private key, and calculate $w$ and $w$ respectively; Both parties choose their own random numbers $a$ and $w$; Finally, exchange $w$ and $w$ with each other • (8) Each party encode plaintext $w$ and $q$ to point $W$ and point $Q$ on $W$ respectively • (9) Alice selects $P(O,P){ = }0$ random numbers $P(O,P){ = }0$($P(O,P){ = }0$) to compute $P(O,P){ = }0$. Bob selects $P(O,P){ = }0$ random numbers $P(O,P){ = }0$($P(O,P){ = }0$) and computes $P(O,P){ = }0$. Finally, the two sides exchanged $P(O,P){ = }0$ and $P(O,P){ = }0$ with each other • (10) Alice randomly selects the $P(O,P){ = }0$ group from the received $P(O,P){ = }0$ group $P(O,P){ = }0$ by using the cut-choose method, and informs Bob that Bob will send the corresponding $P(O,P){ = }0$ to Alice. Alice verifies and calculates it after receiving it: $P(O,P){ = }0$. If the verification is correct, continue the protocol, otherwise stop the protocol. Bob randomly selects the $P(O,P){ = }0$ group from the received $P(O,P){ = }0$ group $P(O,P){ = }0$ by using the cut-choose method, and informs Alice that Alice will send the corresponding $P(O,P){ = }0$ to Bob. Bob will verify and calculate it after receiving it: $P(O,P){ = }0$. If the verification is correct, continue the protocol, otherwise stop the protocol • (11) Alice and Bob randomly select one group $P(O,P){ = }0$ and $P(O,P){ = }0$ from the remaining $P(O,P){ = }0$ and $P(O,P){ = }0$ respectively. Meanwhile, Alice selects two random numbers $a$, $p_{1}$. Bob selects two random numbers $b$, $p_{2}$. Alice computes $P(O,P){ = }0$, $P(O,P){ = }0$, $P(O,P){ = }0$. Bob computes $P(O,P){ = }0$, $P(O,P){ = }0$, $P(O,P){ = }0$. And then, the two sides exchanged $P(O,P){ = }0$ and $P(O,P){ = }0$ with each other • (12) After each party receives the information from the other party, Alice computes $P(O,P){ = }0$ and $P(O,P){ = }0$, and sends them to Bob. Bob computes $P(O,P){ = }0$ and $P(O,P){ = }0$, and sends them to Alice • (13) Alice judges ${m_a} \underline{\underline ?} {w_a} - {\lambda _a}$, that is, whether Bob is multiplied private key $P(O,P){ = }0$ and $P(O,P){ = }0$ to obtain $P(O,P){ = }0$ (zero-knowledge proof); At the same time, Bob judges $m_{a} {\kern 1pt} {\kern 1pt} \underline{\underline{?}} {\kern 1pt} {\kern 1pt} w_{a} - \lambda_{a}$, that is, whether Alice is really multiplies her private key $P(O,P){ = }0$ and $P(O,P){ = }0$ to obtain $P(O,P){ = }0$ (zero knowledge proof). The failed party has malicious behavior • (14) Alice can get $P(O,P){ = }0$ by calculating $P(O,P){ = }0$. If $P(O,P){ = }0$, then $P(O,P){ = }0$; Bob can get $P(O,P){ = }0$ by calculating $P(O,P){ = }0$. If $P(O,P){ = }0$, then $P(O,P){ = }0$. If $P(O,P){ = }0$, it is proved that the results computed by both parties are correct, then output $P(O,P){ = }0$; otherwise, output $P(O,P){ = }1$. Similarly, it can be judged whether another vector $Z^{\prime}_{2} = (b^{\prime}_{1} ,b^{\prime}_{2} ,...,b^{\prime}_{{m_{b} }} )$ of Bob is similar to the vector $Z_{1} = {(}a_{1} ,a_{2} ,...,a_{{m_{a} }} {)}$ of Alice **The protocol ends**	

### Correctness analysis

- The ciphertext sent to each other in steps (1) and (2) does not disclose information, because both parties have added their own random numbers $r_{i}$ and $r_{i}^{\prime }$.
- In steps (3) and (4), Alice and Bob perform homomorphic operations respectively:
  18 $$\begin{aligned} (c_{{b_{1} }} )^{\mu } & = (1 + n^{\prime})^{{\mu }{b_{i} }} r_{i}^{n \prime \mu } \bmod n^{{\prime}{2}} \\ & = (1 + \mu n^{\prime})^{{b_{i} }} r_{i}^{n \prime \mu } \bmod n^{{\prime}{2}} \\ \end{aligned}$$
  19 $$\begin{aligned} (c_{{a_{1} }} )^{\omega } &= (1 + n)^{{\omega }{a_{i} }} r_{i}^{n \omega } \bmod n^{2} \\ & = (1 + \omega n)^{{a_{i} }} r_{i}^{n \omega } \bmod n^{2} \\ \end{aligned}$$
- In steps (5) and (6), Alice and Bob decrypt the calculation using their own private keys $\lambda_{1}$ and $\lambda_{2}$ respectively:
  20 $$\begin{gathered} w = \frac{{\prod\limits_{i = 1}^{{m_{a} + k_{a} + k_{b} }} {L({{{\rm c}^{\prime}}}_{{a_{T(i)} }}^{{\lambda_{1} }} \bmod n^{2} )} }}{{\prod\limits_{i = 1}^{{m_{a} + k_{a} + k_{b} }} {L(\overline{{\text{c}}}_{{b_{T(i)} }}^{{\lambda_{1} }} \bmod n^{2} )} }} \\ = \prod\limits_{i = 1}^{{m_{a} + k_{a} + k_{b} }} {\frac{{{(}(1 + \omega n)_{{}}^{{a_{i} \tau_{i} }} r_{i}^{n\omega } \bmod n^{2} - 1)/n}}{{{(}(1 + \omega n)^{{b_{i} \eta_{i} }} r_{i}^{n\omega } \bmod n^{2} - 1)/n}}} \\ = \prod\limits_{i = 1}^{{m_{a} + k_{a} + k_{b} }} {\frac{{1 + a_{i} \tau_{i} \omega n - 1}}{{1 + b_{i} \eta_{i} \omega n - 1}}} \bmod n^{2} \\ = \prod\limits_{i = 1}^{{m_{a} + k_{a} + k_{b} }} {\frac{{a_{i} \tau_{i} }}{{b_{i} \eta_{i} }}} \bmod n^{2} \\ \end{gathered}$$
  21 $$\begin{gathered} q = \frac{{\prod\limits_{i = 1}^{{m_{b} + k_{a} + k_{b} }} {L(\overline{{\text{c}}}_{{a_{T(i)} }}^{{\lambda_{2} }} \bmod n^{{\prime}{2}} )} }}{{\prod\limits_{i = 1}^{{m_{b} + k_{a} + k_{b} }} {L({{{\rm c}^{\prime}}}_{{b_{T(i)} }}^{{\lambda_{2} }} \bmod n^{{\prime}{2}} )} }} \\ = \prod\limits_{i = 1}^{{m_{b} + k_{a} + k_{b} }} {\frac{{{(}(1 + \mu n^{\prime})_{{}}^{{a_{i} \tau_{i} }} r_{i}^{{n^{\prime}\mu }} \bmod n^{{\prime}{2}} - 1)/n^{\prime}}}{{{(}(1 + \mu n^{\prime})^{{b_{i} \eta_{i} }} r_{i}^{{n^{\prime}\mu }} \bmod n^{{\prime}{2}} - 1)/n^{\prime}}}} \\ { = }\prod\limits_{i = 1}^{{m_{b} + k_{a} + k_{b} }} {\frac{{1 + a_{i} \tau_{i} \mu n^{\prime} - 1}}{{1 + b_{i} \eta_{i} \mu n^{\prime} - 1}}} \bmod n^{{\prime}{2}} \\ = \prod\limits_{i = 1}^{{m_{b} + k_{a} + k_{b} }} {\frac{{a_{i} \tau_{i} }}{{b_{i} \eta_{i} }}} \bmod n^{{\prime}{2}} \\ \end{gathered}$$
- In step (8), plaintexts are encoded to the points $Z_{1} = {(}a_{1} ,a_{2} ,...,a_{{m_{a} }} {)}$ and $Z_{1} = {(}a_{1} ,a_{2} ,...,a_{{m_{a} }} {)}$ of elliptic curve, and the encoding method can refer to Reference^37^.
- The $Z_{1} = {(}a_{1} ,a_{2} ,...,a_{{m_{a} }} {)}$ and $Z_{1} = {(}a_{1} ,a_{2} ,...,a_{{m_{a} }} {)}$ exchanged in step (9) do not have data leakage, because of the addition of their own random numbers.
- In step (11), Alice and Bob compute respectively:
  22 $$\begin{aligned} c_{b} & = a(c_{2b}^{j} - c_{1b}^{j} - W + K_{2} ) \\ & = a(Q + f_{j} Q + bG - f_{j} Q - K_{2} - W + K_{2} ) \\ & = a(Q - W) + abG \\ \end{aligned}$$
  23 $$\begin{aligned} c_{a} & = b(c_{2a}^{i} - c_{1a}^{i} - Q + K_{1} ) \\ & = b(W + d_{i} W + aG - d_{i} W - K_{1} - Q + K_{1} ) \\ & = b(W - Q) + abG \\ \end{aligned}$$

Then Alice and Bob send $Z_{1} = {(}a_{1} ,a_{2} ,...,a_{{m_{a} }} {)}$ and $Z_{1} = {(}a_{1} ,a_{2} ,...,a_{{m_{a} }} {)}$ to each other.

- The final result obtained by Alice and Bob in step (14) is correct because:

Alice uses the zero-knowledge proof to verify the $Z_{1} = {(}a_{1} ,a_{2} ,...,a_{{m_{a} }} {)}$ sent by Bob, and the result obtained by calculating $Z_{1} = {(}a_{1} ,a_{2} ,...,a_{{m_{a} }} {)}$ is correct, that is:

24 $$\begin{aligned} m_{b} - av & = m_{b} - abK_{2} \\ &= k_{2} c_{b} - abk_{2} G \\& = k_{2} a(Q - W) + k_{2} abG - abk_{2} G \\& = k_{2} a(Q - W) \\ \end{aligned}$$

Bob uses the zero-knowledge proof to verify the $Z_{1} = {(}a_{1} ,a_{2} ,...,a_{{m_{a} }} {)}$ sent by Alice. The result obtained by calculating $Z_{1} = {(}a_{1} ,a_{2} ,...,a_{{m_{a} }} {)}$ is correct, that is:

25 $$\begin{aligned} m_{a} - bu & = m_{a} - baK_{1} \\ &= k_{1} c_{a} - bak_{1} G \\ & = k_{1} b(W - Q) + k_{1} baG - bak_{1} G \\ & = k_{1} b(W - Q) \\ \end{aligned}$$

- At the end of the protocol, there is no data leakage, and both parties can get the correct result, and the final result of the protocol is calculated by both parties, which is fair.

### Security proof

In Protocol 2, Alice and Bob have the same steps to implement the protocol. We take the possible malicious behaviors of Alice as an example to analyze the security of Protocol 2:

If the $d_{i}$ selected by Alice in step (9) is the wrong random number, Bob happens not to pick the wrong random number $d_{i}$ in $\frac{m}{{2}}$, that is, the wrong random number $d_{i}$ is not detected, and it happens to be selected by Bob in step (11), and finally Bob computes the wrong result. If Alice implements malicious attacks, the case where the probability of successful implementation is the highest is that Alice mixes $m$ random numbers $a_{i}$ with one wrong $a_{i}$, so the probability of successful implementation of this malicious behavior is the highest. In this case, the probability of successful deception is $\frac{1}{m}$. If $m = 20$ and Alice mixes $m$ random numbers $a_{i}$ with one wrong $a_{i}$, the probability of successful deception in this case is $\frac{{C_{10}^{10} }}{{C_{20}^{10} }} \times \frac{1}{10} = \frac{1}{200}$, but if Alice mixes $m$ random numbers with ten wrong $a_{i}$, the probability of successful deception in this case is $\frac{{C_{10}^{10} }}{{C_{20}^{10} }} \times \frac{1}{2} = 2.7 \times 10^{ - 7}$, and the probability of success in this case is smaller to zero. If Alice mixes more than $\frac{m}{{2}}$ wrong random number $a_{i}$ into $m$ random number $a_{i}$, it will be found in the subsequent verification stage. Therefore, our protocol is secure under this malicious behavior.

#### Theorem 1

*Under the malicious model, Protocol 2 is secure.*

#### Proof

To prove that the protocol $\prod$ is secure in the malicious model, we must be able to convert the acceptable policy pair $\overline{A} = (A_{1} ,A_{2} )$ in the real model protocol when executing $\prod$ into the corresponding policy pair $\overline{B} = (B_{1} ,B_{2} )$ in the ideal model protocol, so that the output calculation of $A_{1} ,A_{2}$ in $\prod$ cannot be distinguished from that of $B_{1} ,B_{2}$ in the ideal protocol. Because both parties are not allowed to be dishonest at the same time in the malicious model, we deal with the two cases: $A_{1}$ is honest or $A_{2}$ is honest, respectively.

(1) $$A_{1}$$ honest, $$A_{2}$$ dishonest:

26 $$REAL_{{\overline{A} }} (W,Q) = \{ F(W,A_{2} (Q)),A_{2} ((c_{1a}^{i} ,c_{2a}^{i} ),m_{a} ,S)\}$$

Because $A_{1}$ is honest, $B_{1}$ will transmit the correct $W$; However, the choice of $B_{2}$ depends on $A_{2}$. Ideally, $B_{2}$ will send $Q$ to $A_{2}$. In fact, $A_{2}$ will send $A_{2} (Q)$ to $B_{2}$. The final protocol will output $F(W,A_{2} (Q))$. Ideally, $B_{2}$ will make its own $F(W,A_{2} (Q))$ indistinguishable from the $view_{{A_{2} }} (W,A_{2} (Q))$ of $A_{2}$ in the real situation, that is, select $W^{\prime}$ to get $F(A_{1} (W),Q^{\prime}) = F(A_{1} (W),Q)$, and after completing the protocol, it will get $m^{\prime}_{a}$ $c^{\prime}_{1a} ,c^{\prime}_{2a}$ and $S^{\prime}$ obtained by zero-knowledge proof:

27 $$\{ IDEAL_{{\overline{B} }} (W,Q)\} = \{ F(W,A_{2} (Q)),A_{2} ((c_{1a}^{{i^{\prime}}} ,c_{2a}^{{i^{\prime}}} ),m^{\prime}_{a} ,S^{\prime})\}$$

Because the same encryption tools and probability algorithms are used, $c_{1a}^{{i{\prime}}} { \equiv }^{\!\!\!\!\!C} c_{1a}^{i}$, $c_{2a}^{{i{\prime}}} { \equiv }^{\!\!\!\!\!C} c_{2a}^{i}$, therefore:

28 $$\{ REAL_{{\overline{A} }} (W,Q)\} = \{ IDEAL_{{\overline{B} }} (W,Q)\}$$

(2) When $A_{1}$ is dishonest and $A_{2}$ is honest, the real situation can be divided into two types:

- $A_{1}$ complete the zero-knowledge proof and publish its results:
  29 $$\{ REAL_{{\overline{A} }} (W,Q)\} = \{ A_{1} ((c_{1b}^{i} ,c_{2b}^{i} ),m_{b} ,S),F(W,Q)\}$$
- $A_{1}$ does not conduct zero-knowledge proof or publish its results:
  30 $$\{ REAL_{{\overline{A} }} (W,Q)\} = \{ A_{1} ((c_{1b}^{i} ,c_{2b}^{i} ),m_{b} ,S), \bot \}$$

Since $A_{2}$ is honest, $B_{2}$ will transmit the correct $Q$; However, the choice of $B_{1}$ depends on $A_{1}$. Ideally, $B_{1}$ will send $W$ to $A_{1}$, in fact, $A_{1}$ will send $A_{1} (W)$ to $B_{1}$, and the final protocol output is $F(A_{1} (W),Q)$. If $A_{1}$ does not prove zero-knowledge or publish the results in practice, $B_{2}$ will ideally get $\bot$. Ideally, $B_{1}$ will make its own $F(A_{1} (W),Q)$ indistinguishable from the $view_{{A_{1} }} (A_{1} (W),Q)$ of $A_{1}$ in the real situation, that is, select $Q^{\prime}$ to get $F(A_{1} (W),Q^{\prime}) = F(A_{1} (W),Q)$, and after completing the protocol, get $m^{\prime}_{b}$, $c_{1b}^{{i{\prime}}} ,c_{2b}^{{i{\prime}}}$ and $S^{\prime}$ obtained by zero-knowledge proof:

- Ideally, when $B_{1}$ does not publish the results:
  31 $$IDEAL_{{\overline{B} }} (W,Q) = \{ A_{1} ((c_{1b}^{{i{\prime}}} ,c_{2b}^{{i^{\prime}}} ),m^{\prime}_{b} ,S^{\prime}), \bot \}$$
- Ideally, when $B_{1}$ publishes the results:
  32 $$IDEAL_{{\overline{B} }} (W,Q) = \{ A_{1} ((c_{1b}^{{i{\prime}}} ,c_{2b}^{{i^{\prime}}} ),m^{\prime}_{b} ,S^{\prime}),F(A_{1} (W),Q)\}$$

$c_{1b}^{{i{\prime}}} ,c_{2b}^{{i{\prime}}}$ and $c_{1b}^{i} ,c_{2b}^{i}$ use the same cryptosystem, and $m^{\prime}_{b}$ and $m_{b}$ are also operations between constants. Zero-knowledge proof ensures the correctness of $S^{\prime} { \equiv }^{\!\!\!\!\!C} S$, so there are:

33 $$IDEAL_{{\overline{B} }} (W,Q) { \equiv }^{\!\!\!\!\!C} REAL_{{\overline{A} }} (W,Q)\}$$

The security of Protocol 2 under the malicious model is proved.

## Efficiency analysis

This paper compares and analyzes the efficiency of Protocol 2 with existing references^17,26^^,^^27^.

### Computational complexity

Reference^17^ proposed a MPC protocol of graphic similarity based on hash operations, and carried out hash operations for 6 times, but it can not resist the attack of malicious enemies. Reference^26^ also carried out $6\log N$ modular multiplications based on the rational number equality MPC protocol under the malicious model designed by ECC, but the two sides of the protocol are not fair, and the protocol can only resist some malicious attacks. Reference^27^ solves the problem of determining the position relationship between two rational intervals by using the inner product of integer vector, and performs $36\log N$ modular multiplications, but it can not resist malicious behavior.

During the execution of Protocol 2, there are two ECC encryption operations and a total of $(3m_{a} + 3m_{b} + 4k_{a} + 4k_{b} + 2)\log N$ modular multiplication operations (where $m_{a}$, $m_{b}$ is the length of each vector and $k_{a}$, $k_{b}$ is the random number selected by each party). The rest computations are ordinary multiplication and addition operations, which can be ignored.

### Communication complexity

Reference^25^ carried out four rounds of communication. Reference^34^ conducted four rounds. Reference^35^ carried out four rounds. Protocol 2 carried out seven rounds of communication. Table 1 below shows the overall performance comparison of each protocol.

**Table 1:** The overall performance comparison of each protocol.

Protocol	Fair	Computational complexity (modular multiplication)	Communication rounds	Resist malicious adversaries
Reference^25^	No	6 Hash	3	×
Reference^34^	No	$6\log N$	4	√
Reference^35^	No	$36\log N$	4	×
Protocol 2	Yes	$(3m_{a} + 3m_{b} + 4k_{a} + 4k_{b} + 2)\log N$	7	√

Protocol 2 is proposed to solve the graphic similarity secure judgment problem, and can resist the attacks of malicious enemies. Therefore, it is not comparable with the existing graphic isomorphic secure judgment protocol under the semi-honest model in terms of efficiency. In addition, for the MPC protocol of malicious participants, the cut-choose method and zero-knowledge proof will greatly increase the computational complexity and significantly reduce the execution efficiency, making the computational efficiency of the anti-malicious MPC protocol lower than that of the semi-honest MPC protocol. However, computing outsourcing or preprocessing can be used to improve the execution efficiency of the protocol.

In the case of little difference in communication rounds, Protocol 2 adopts relatively simple and fast ECC encryption, which improves the calculation efficiency, and can securely and effectively prevent the occurrence of malicious behavior, making our protocol more secure and widely used.

### Experimental simulation

To make our protocol more intuitively show its efficiency, we compare Protocol 2 with Reference^25^, Reference^34^, and Reference^35^. The experimental environment is windows10 (64 bit) operating system, Intel (R) core (TM) i7-5500u CPU @ 2.40 GHz processor and 8.00gb memory. The specific experiment uses Python language.

**Figure 7:**
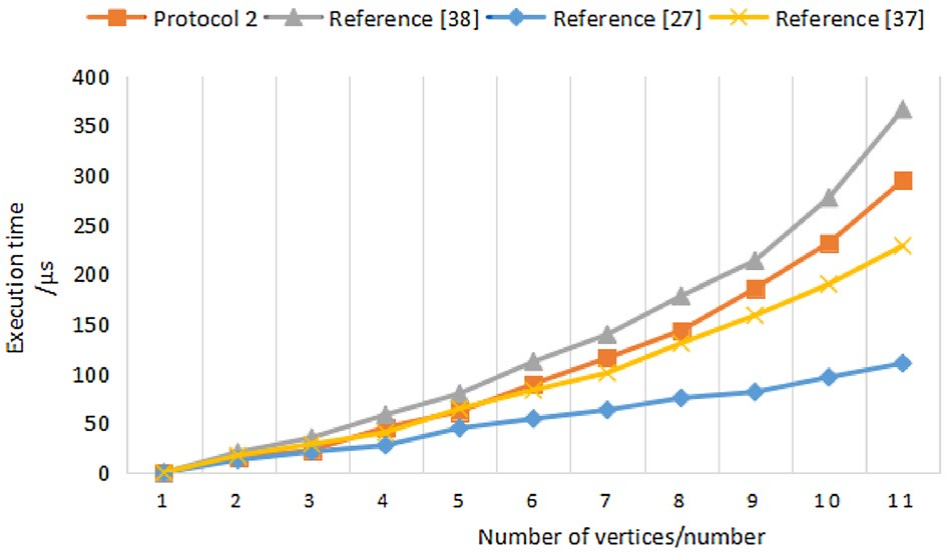
Comparison of the execution time of three protocols.

Two similar graphics are randomly selected, and the number of vertices of similar graphics increases from 0 to 500. Experiments are carried out on two similar graphics with vertex points of 50, 100, 150, 200, 250, 300, 350, 400, 450, and 500 respectively by using the above four protocols (rational numbers in Reference^34^ and Reference^35^ are equivalent to vectors in similar graphics). In Fig. 7, the ordinate represents the time consumed (μs), and the abscissa represents the number of different vertices.

Through the comparison with the experiment in Reference^25^, it is found that the security and accuracy of using hash function anti-collision as the graphic isomorphism confidentiality judgment protocol can be further improved. The hash function is usually used in information authentication and digital signature, while in Reference^25^, the hash function is used to map fixed-length graphic information to another certain length information, in which it is difficult to resist the attacker's violent cracking of graphic private information. Compared with References^34^^,^^35^, Protocol 2 has no big difference in time performance consumption in the case of the small amount of data, but its security performance is improved. Protocol 2 has higher security and practical values.

## Applications

With the rapid development of geometric image retrieval technology, the judgment of graphic similarity gradually appears, which has been widely used in the fields of terrain matching, mechanical parts matching, biomolecule, and face recognition. Therefore, it is very necessary to study the secure judgment of graphic similarity.

In the field of terrain matching, it is particularly important to make a correct judgment in time when the ship is sailing in the face of unknown glaciers, and other terrains, and when the aircraft is flying in the face of unknown obstacles. This protocol can be used to judge whether the terrain is matched in advance. Due to the difficulty of oilfield development, security problems and terrain reasons, the calculation of oilfield similarity have always been a problem for oil explorers. Using the protocol in this paper, we can judge the oilfield similarity by combining theoretical knowledge with practice in a virtual scene.

**Figure 8:**
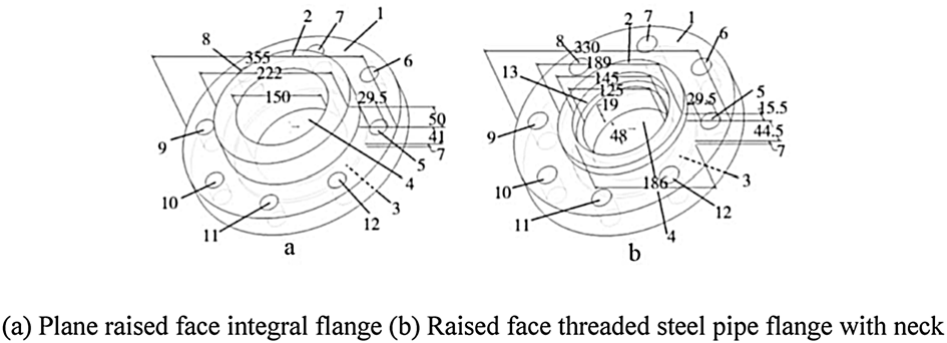
Matching judgment of flange parts.

For the mechanical parts used in military and national defense, if you do not want to disclose patent data, how to match firmly, you need to judge the graphic similarity under the condition of confidentiality. Using this protocol to analyze the similarity of parts, the parts in new products can share some design parameters in the design and manufacturing stage. Through the replacement and merging of parts, the reuse of parts can be improved and the redundant parts can be reduced. Similar flange parts as shown in Fig. 8 (figure (a) is a plane raised face integral flange, figure (b) is a raised face threaded steel pipe flange with neck).

**Figure 9:**
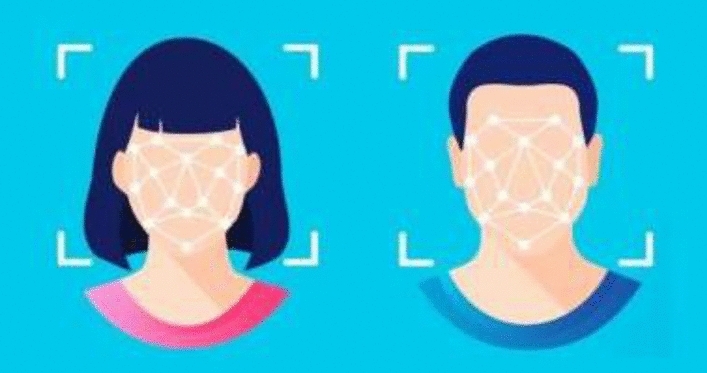
Face recognition matching technology.

In addition, face recognition, network image fuzzy search can use Protocol 2 to execute. With the explosive growth and easy access of image sources, face recognition has become more and more important. The disclosure of personal privacy information has also attracted people's attention to face recognition technology. The face recognition system mainly extracts the main geometric feature points of the face (such as face contour), the continuous shape of the main organs of the face, the curvature of geometric features, etc., as shown in Fig. 9 below.

## Conclusion

The secure judgment of graphic similarity is always an important problem in secure multi-party computing geometry. Using the Paillier variant cryptosystem, ECC, zero-knowledge proof and cut-choose methods, aiming at the possible malicious attacks of the semi-honest model protocol, this paper proposes an MPC protocol of graphic similarity under the malicious model. Finally, the real/ideal paradigm is used to strictly prove that the protocol is secure under the malicious model, can resist the attack behaviors of malicious attacks. In the future, by applying the protocols to the fields of terrain matching, mechanical parts, biomolecules and face recognition, life will be more convenient and fast, with great practical value and significance.

## Data Availability

All data generated or analyzed during this study are included in this paper.
